# The habitat of *Coccidioides* spp. and the role of animals as reservoirs and disseminators in nature

**DOI:** 10.1186/s12879-016-1902-7

**Published:** 2016-10-10

**Authors:** María del Rocío Reyes-Montes, María Ameyali Pérez-Huitrón, Jorge Luis Ocaña-Monroy, María Guadalupe Frías-De-León, Erick Martínez-Herrera, Roberto Arenas, Esperanza Duarte-Escalante

**Affiliations:** 1 Departamento de Microbiología y Parasitología, Facultad de Medicina, Universidad Nacional Autónoma de México (UNAM), Ciudad Universitaria No. 3000, Coyoacán, 04510 México Cd.Mx., Mexico; 2 División de Investigación, Hospital Juárez de México, Edificio E. Av. Instituto Politécnico Nacional 5160, Col. Magdalena de las Salinas, 07760 México Cd.Mx., Mexico; 3 Unidad de Micología, Hospital General “Manuel Gea González”, Av. Calzada de Tlalpan 4800, Tlalpan, 14080 México, Cd.Mx., Mexico

**Keywords:** *Coccidioides* spp., Reservoir, Habitat, Dissemination

## Abstract

**Background:**

Coccidioidomycosis, a potentially fatal fungal infection, is considered an emergent mycotic disease because of the increased incidence of fungal infections registered over recent years. Infection occurs through the inhalation of arthroconidia from two main species of *Coccidioides*: *Coccidioides immitis* and *C. posadasii*, which are both endemic to arid and semi-arid regions of North America. *Coccidioides* species not only infect humans but can also infect other mammals (land, aquatic, wild or domestic), reptiles and birds.

**Objective:**

To obtain information regarding the habitat of *Coccidioides* spp. and the animals infected by this fungus and to identify the role that infected animals play as reservoirs and disseminators of this fungus in nature.

**Materials:**

A literature review was conducted to identify the habitat of *Coccidioides* spp. and the infected non-human animal species targeted by this fungus.

**Results and conclusions:**

This review allows us to suggest that *Coccidioides* spp. may be classified as halotolerant organisms; nevertheless, to perpetuate their life cycle, these organisms depend on different animal species (reservoirs) that serve as a link with the environment, by acting as disseminators of the fungi in nature.

## Background

Coccidioidomycosis, a potentially fatal fungal infection, is considered an emergent mycotic disease because of the increased incidence of infections registered in recent years [1]. Infection occurs through the inhalation of arthroconidia from two species of *Coccidioides*: *Coccidioides immitis* and *C. posadasii* [2], which are both endemic to arid and semi-arid regions of North America. The zones most affected by this mycosis are endemic regions of the southwestern United States of America (USA), northern Mexico, Central America and some regions of South America [3, 4]. *Coccidioides* spp. inhabit the soil throughout these endemic regions; therefore, understanding the habitat occupied by this fungus is important to identify the epidemiology of the mycotic human disease. However, coccidioidomycosis not only occurs in humans but may also affect several animal species, a fact that has not received much attention but nonetheless relevant for establishing the role of animals as vectors in the epidemiology of this disease in humans [5].

The objective of this study was to review the information in the literature regarding the habitat of *Coccidioides* spp. and the animal species affected by coccidioidomycosis. This information may aid in elucidating the role of infected animal species as reservoirs and disseminators of disease.

## Discussion

### Habitat of *Coccidioides* spp.

Regions that are endemic for coccidioidomycosis are generally characterized by a semi-arid climate that facilitates the spread of *Coccidioides* spores in the air. The mild winter weather typical of endemic regions provides optimal conditions for the growth of the fungus, and the dry, hot summers facilitate the dispersion of the arthroconidia [3, 6, 7]. However, the distribution of *Coccidioides* spp. in the soil of endemic regions is irregular, even in regions with a high incidence of infection [8].

The first studies to describe the habitat of *Coccidioides* were those of Egebert et al. [9], and Elconin et al. [10] and Lacy and Swatek [11]; those researchers obtained fungal isolates from soil samples with a high salinity and suggested that elevated soil salinity may be a requirement for fungal development. The spores of *Coccidioides* are also known to grow in sandy alkaline soils that are rich in organic matter and salts [7, 12–14]. Fisher et al. [15] confirmed that the habitat of *Coccidioides* in endemic regions is characterized also by soils with high levels of essential nutrients such as iron, calcium and magnesium. Moreover, additional findings from Fisher et al. [15] support the ability of this fungus to grow in nearly any type of desert soil, including those with low pH levels; this fungus can also tolerate extreme air temperatures ranging from -40.0 to 48.8 °C and soil temperatures ranging from -6.5 to 60.5 °C. The authors also emphasize that abiotic factors in endemic zones may provide favourable conditions for the growth of *Coccidioides*. Greater organic content in the soil may lead to a greater availability of nutrients for fungal growth, whereas increased salinity and high temperatures may decrease the competition with other microorganisms. For example, high concentrations of sodium borate in the soil may be antiseptic to some microorganisms but not necessarily to *Coccidioides* [15]. More recently, work by De Macedo et al. [16] confirmed the environmental characteristics described previously for the habitat of *Coccidioides* spp. through the positive identification of *Coccidioides* isolates in soil samples obtained from semi-arid regions in the state of Piauí, in the northeastern region of Brazil.

Dabrowa et al. [17] determined the presence of potentially pathogenic fungi in intertidal zones. Although the authors were unable to confirm positive isolates of *C. immitis*, it is possible that this fungus may be present in intertidal zones considering the endemic presence of *Coccidioides* throughout the southwestern USA. Moreover, it was previously demonstrated that *C. immitis* is able to survive in seawater and saturated salt water for up to 6 weeks in the laboratory [18]. Importantly, even under these conditions, *C. immitis* maintains the ability to infect various aquatic species such as river crabs, goldfish, bottle nose dolphins and lion fish [18]. The concentration of *C. immitis* in these sediments is naturally elevated during the rainy season as the result of ocean or river runoff [3]. In Mexico, despite successful clinical isolations of *Coccidioides* spp., the screening of environmental samples has had low effectiveness, so that Catalán-Dibene et al. [19] explored a highly endemic area near the USA and Mexico border, where previously detected *Coccidioides* by molecular methods [20]. In this study they tested the serum of 40 trapped rodents using ELISA, and detected antibodies against *Coccidioides* in two species: *Peromyscus maniculatus* and *Neotoma lepida*. This study sets the basis for analyzing this pathogen in its natural environment.

Baptista-Rosas et al. [21] generated a model for the distribution of *Coccidioides* species by using a combination of several environmental variables and geospatial reference points in the USA and Mexico, which are places with positive *Coccidioides* isolates. This model suggests that arid soils in North American deserts are likely to serve as ecological niches for *Coccidioides*, in agreement with the greater incidence of disease in these regions and findings by others [9–11].

Currently, findings by various studies agree regarding the ecological traits that define the habitat of *Coccidioides*. This type of fungus is associated with soils in arid, semi-arid and alkaline regions that have sparse xerophytic vegetation, elevated average temperatures and a low annual average rainfall [15]. Therefore, the ability of *Coccidioides* to survive under such extreme conditions represents an advantage over other microorganisms, as suggested by Baumgardner [5]. Based on work by De Hoog [22], the *Coccidioides* genus consists of xerotolerant fungi because they can grow under extreme hostile conditions. Moreover, *Coccidioides* may be considered halotolerant organisms [23], because both species in this genus tolerate high salt concentrations of up to 8 % [2, 24, 25]. Nevertheless, little is known about the mechanisms that species of *Coccidioides* utilize to survive under high salinity conditions. Some strategies that *Coccidioides* may utilize to survive under these conditions may include: (1) maintaining high intracellular salt concentrations, osmotically or at least equivalent to its external concentration (“salt-in” strategy) allowing for it to adapt to an alkaline environment. However, this requires a special adaptation of its intracellular systems; and (2) maintaining low salt concentrations in its cytoplasm (“compatible-solute” strategy). The osmotic pressure of the medium is balanced by compatible solutes (2-sulfotrehalose, trehalose, saccharose, glycerol, betaine glycine, ectoin and glycosilglycerol). These solutes do not affect the enzymatic activity as high concentrations of inorganic salts do not require a special adaptation of intracellular systems [23]. However, it remains to be determined which strategies are used by *Coccidioides* to survive under conditions of high salinity.

### Role of animals as reservoirs

It has been suggested that animals may play a role in soil enrichment by serving as a substrate or growth factor for some pathogens [5]. For example, the growth of *Histoplasma capsulatum* or *Cryptococcus neoformans* is favoured in soils rich in animal-derived excrements and organic matter [5].

Authors have proposed for more than five decades that animal carcasses may serve as a medium for the growth of *Coccidioides* in the soil [26–28]. This was supported by the finding of fungus-positive isolates near animal burrows [29–31], in contrast, isolates from soil without animal contact were negative for the fungus [32]. It is important to emphasize that although some work suggests the existence of “hot spots” in endemic zones or areas where the presence of *Coccidioides* is highly probable [21], attempts to isolate the fungus from the soil have been unsuccessful. Nonetheless, coccidioidomycosis has typically been considered a “classical” soil-acquired infection [5]. However, work by [24] emphasized the role of animals in the life cycle of *Coccidioides*, with the finding of fungus-positive isolates in samples collected from animal burrows in the city of Solonolpoles in the state of Ceara, in northeastern Brazil. This city is located at an elevation of 155. 38 metres above sea level, has an average temperature of 26–28.8 °C, has a rainy season from January to April with an average annual precipitation of 700 mm and contains xerophytic vegetation. Solonolpoles is located in a semi-arid region of Brazil and is characterized by high temperatures, frequent drought and friable soil. These characteristics are in agreement with those previously described for the habitat of *Coccidioides* spp. and provide further support to the idea that animals may enrich the soil and act as an important growth factor for this fungus [5]. Additionally, it has been proposed that infected animals, such as bats and armadillos, may form part of the life cycle of *Coccidioides* and thus act as reservoirs of the fungus [30, 33, 34].

Recent findings from comparative genomic analyses suggest that *Coccidioides* spp. contain a significant number of genes that are important for host interactions, and the number of genes involved in host interactions is greater than those important for survival in their natural habitat [35]. This trend is further supported by the finding of numerous changes in the genetic makeup of *Coccidioides*, with the biggest change occurring in the number of genes that are important for survival in natural habitats. For example, the decrease of genes involved in cell wall degradation (cellulase, tannase, cutinase and pectin lyase) occurs together with an expansion in the number of genes involved in the use of carcasses as an energy source (protease and keratinase). Some of these genes encode protease families, such as extracellular serine proteases, aspartic proteases, and Meps. At least ten Mep genes (designated as Mep1 to Mep10) have been found in *Coccidioides posadasii*, and most of them were classified into the M35 (deuterolysin) and M36 (fungalysin) families [36]. In sum, these changes suggest that *Coccidioides* spp. are not typical soil fungi in that they maintain a close association with keratin-rich animals both during the infection of a living host and after the host has died by growing in the carcass as mycelium [35, 37].

**Fig. 1:**
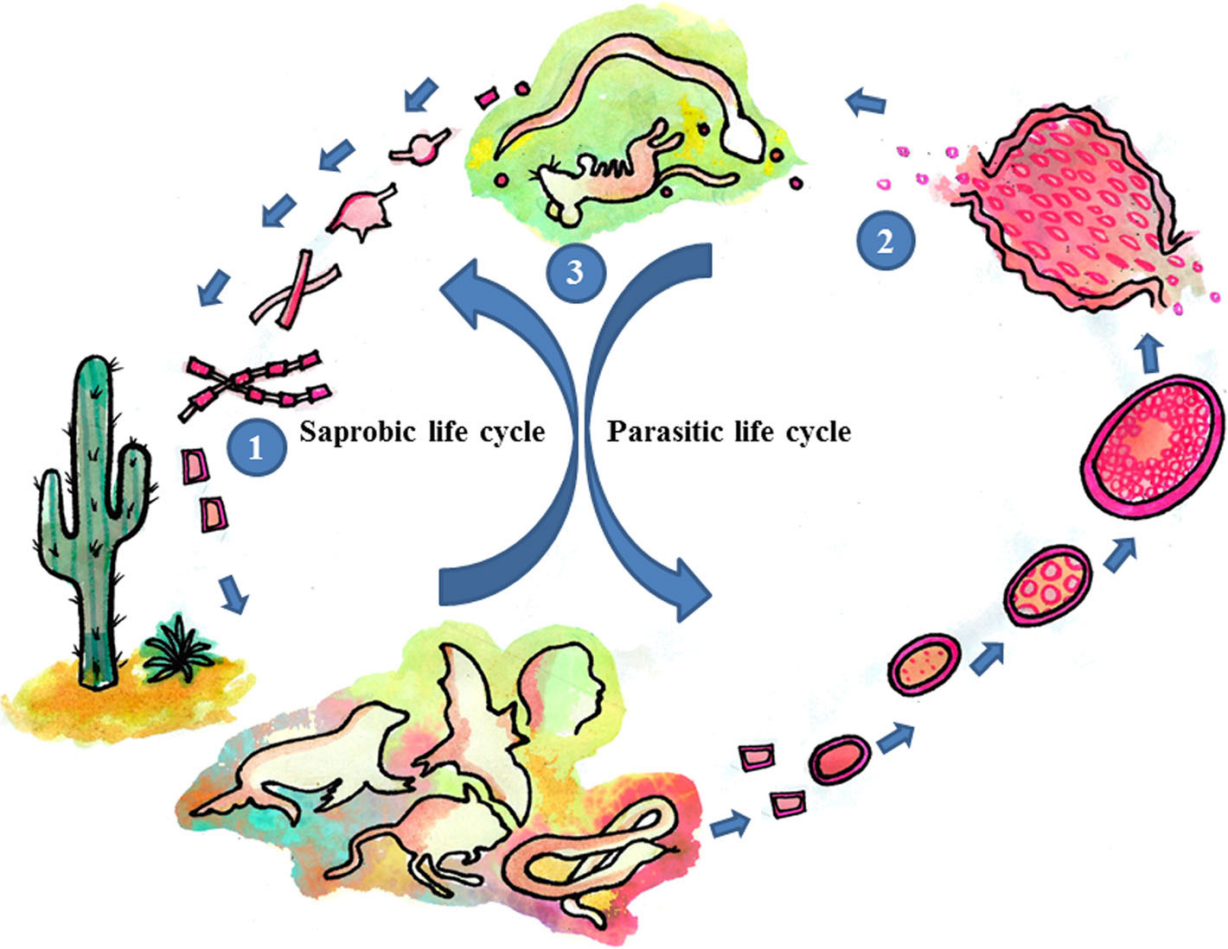
Fungi belonging to the genus *Coccidioides* inhabit arid and semi-arid zones with alkaline soils and extreme temperatures. Under these conditions, they grow in a mycelial form and develop asexual reproductive structures. **(1)** These structures may be dispersed by the wind and find a host, primarily mammals, including humans, where dimorphic changes occur, giving rise to a parasitic form that has spherules and endospores. **(2)**, Hundreds of endospores are released, and each endospore has the capacity to give rise to another spherule, repeating the life cycle in the host. In humans, the infection may progress to disease, or it may be eradicated by the immune system. Similarly, in animals, the infection may or may not lead to disease. In the case of disease and eventual death of the host, *Coccidioides* become exposed to the environment and return to a mycelial form **(3)**, thus becoming integrated once again into their habitat

Overall, these findings may suggest that the location of *Coccidiodes* spp. in the environment is intricately tied to the activities of their host. The low number of fungus isolates from soil samples appears to agree with the observation that *Coccidiodes* are better adapted to a life cycle that includes an animal host. In addition to the lower number of fungus isolates that can be obtained from soil samples, the finding that most of the positive soil isolates are found in association with organic matter derived from animal carcasses further supports intricate host interactions. Therefore, based on these findings, we propose that the life cycle of *Coccidioides* spp. in the natural soil habitat may be brief. Once *Coccidioides* spp. infect a host, disease may progress, leading ultimately to the death of the host and providing new organic matter seeded with fungal particles. The high temperatures in these endemic zones, combined with the elevated levels of carbon dioxide that result from the process of decomposition, provide an optimal environment for the dimorphic transformation of the parasitic structures (spherules) of *Coccidioides*, giving rise to the infective forms (arthroconidia). Upon completion of this life cycle, new infective fungi are available for dispersion by established mechanisms [38] (Fig. 1), thus allowing the fungi to rapidly infect new hosts and re-start the cycle. Therefore, the time spent by the fungus without host interaction is brief, which partly explains the difficulty of obtaining fungus-positive isolates from soil samples (Fig. 1). The brief existence of *Coccidioides* spp. in the absence of a host may be related to the extreme environmental conditions of the ecological niche. The high salt concentrations in the soil or water present an energetically costly challenge to life. Under conditions of high salinity, the fungus must maintain ionic concentration gradients to ensure osmotic equilibrium [39]. The extreme environmental conditions have driven adaptive changes in the genome of *Coccidioides* spp., leading to the acquisition of a small number of new genes that are mostly associated with the interactions between the fungus and their animal hosts [35]. Therefore, these genetic changes ensure the use of animals as reservoirs by *Coccidioides* spp., which appear to provide a favourable environment for fungal development.

The extreme environmental conditions present in the natural soil habitat of *Coccidioides* spp. are not favourable for the sexual reproduction of the fungus because this process requires greater energy expenditure [23]. We suggest that *Coccidioides* spp. tend to reproduce asexually in the natural habitat; in contrast, when the fungus encounters a suitable animal host, sexual reproduction may resume, although sexual reproduction has not been demonstrated in *Coccidioides* spp., there are evidences for this type of reproduction [40–42]. These changes in reproduction modalities according to habitat may partly explain the genetic variability in *Coccidioides* spp. [43].

### Mechanisms for the dissemination of *Coccidioides* spp.

Coccidioidomycosis has been reported in a number of animal species (Table 1); however, to date, the role of animals in the epidemiology of the human disease has not been fully considered.

**Table 1:** Findings of *Coccidioides* spp. in non-human animals

Specie	Diagnostic	Reference
Terrestrial mammals in captivity		
*Panthera tigris tigris* (bengal tiger)	*Post-mortem* (histopathology and serology)	[64]
*Macropus rufus* (kangaroo)	*Post-mortem* (histopathology and serology)	[65]
*Macaca mulatta (*rhesus monkey)	*Post-mortem* (histopathology and serology)	[66]
*Theropithecus gelada* (primate)	*Post-mortem* (histopathology)	[67]
*Lama glama* (llama)	*Post-mortem* (histopathology and serology)	[68]
*Papio cynocephalus* (yellow baboon)	*Post-mortem* (histopathology and serology)	[69]
*Tayassu tacaju* (collared peccary)	*Post-mortem* (histopathology)	[70]
*Tayassu tacaju* (peccary)	*Post-mortem* (histopathology)	[71]
*Lemur catta* (lemur)	*Post-mortem* (serology)	[72]
*Lama glama* (llama)	*Post-mortem* (histopathology, serology and culture)	[73]
*Mandrillus sphinx* (mandrill)	*Post-mortem* (histopathology, serology and culture)	[74]
*Pan troglodytes* (chimpanzee)	*Post-mortem* (histopathology, serology and culture)	[75]
*Phascolarctos cinereus* (koala)	*Post-mortem* (histopathology and serology)	[76]
*Bison antiquus* (bison)	*Post-mortem* (histopathology)	[77]
*Panthera tigris corbetti* (tiger)	*Post-mortem* (histopathology and serology)	[78]
*Pan troglodytes* (chimpanzee)	Histopathology	[79]
*Diceros bicornis* (rhino)	*Post-mortem* (histology and serology)	[80]
*Lama glama* (llama)	*Post-mortem* (histopathology, serology and culture)	[81]
*Nomascus gabriellae* (monkey)	Serology	[82]
*Vicugna pacos* (vicuna)	*Post-mortem* (histopathology, serology and PCR)	[83]
Marine mammals in captivity		
*Zatopilus californianus* (sea lion)	*Post-mortem* (histopathology and culture)	[58]
*Enhydra lutris* (otter)	*Post-mortem* (histopathology)	[84]
*Zalophus californianus* (sea lion)	*Post-mortem* (histopathology and culture)	[59]
*Tursiops truncatus* (dolphin)	*Post-mortem* (histopathology, serology and culture)	[60]
Domestic mammals		
*Ovis aries* (sheep)	*Post-mortem*	[85]
*Sus domestica* (pig)	*Post-mortem* (histopathology)	[86]
*Equus caballus* (mare)	*Post-mortem* (histopathology)	[87]
*Ovis canadensis nelsoni* (ram)	*Post-mortem* (histopathology and culture)	[88]
*Equus caballus* (mare)	*Post-mortem* (histopathology)	[89]
*Equus caballus* (mare)*.*	Histopathology and culture	[90]
*Equus caballus* (mare)	*Post-mortem* (histopathology)	[91]
*Canis lupus familiaris* (dog)	*Post-mortem* (histopathology and culture	[92]
*Equus caballus* (mare)	*Post-mortem* (histology and serology)	[93]
*Canis lupus familiaris* (dog)	Histology and X-ray	[94]
*Canis lupus familiaris* (dog)	Serology and X-ray	[95]
*Equus ferus przewalskii* (horse)	Records and histopathology	[96]
*Canis lupus familiaris* (dog)	Serology	[97]
*Equus caballus* (horse)	Serology and culture	[98]
*Felis silvestris domesticus* (cat)	Serology and culture	[99]
*Canis lupus familiaris* (dog)	*Post-mortem* (histology and serology)	
*Felis silvestris domesticus* (cat)	Histopathology and serology	[61]
Wild mammals		
*Canis latrans* (coyote)	*Post-mortem* (histopathology)	[100]
*Felis concolor* (cougar)	*Post-mortem* (histopathology and culture)	[101]
*Felis concolor* (cougar)	*Post-mortem* (histopathology)	[102]
*Dasypus novemcinctus*	*Post-mortem* (histopathology and culture)	[30]
(armadillo)		
*Glossophaga soricina* and *Desmodus rotundus* (bats)	*Post-mortem* (histopathology and serology)	[33]
*Peromyscus maniculatus* and *Neotoma lepida* (rodents)	*Post-mortem* (serology)	[19]
Reptiles		
*Pituophis melanoleucus affini* (snake)	*Post-mortem* (histopathology and culture)	[103]
*Masticophis flagellum piceus* (snake)	*Post-mortem* (histopathology and serology)	[104]
Birds		
*Gallus gallus domesticus* (chicken)	*Post-mortem* (culture)	[56]

*PCR* polymerase chain reaction

It has been suggested that domestic land animal species and wild animal species may act as disseminators of *Coccidioides* in the environment, for example, as a result of poor practices for carcass disposal. Pathogenic fungi, including *Coccidioides* spp., can grow “in vitro” in the inorganic components of bones. Therefore, carcasses of infected animals may harbour pathogenic *Coccidioides* spp. and serve as an ecological niche or reservoir from which the pathogenic fungus may be easily dispersed by the wind and rapidly transported over long distances [44, 45] (Fig. 1). The wind moving over infected carcasses or bones may generate bioaerosols [46], this is a likely mechanism of dispersion. Another important characteristic of *Coccidioides* spp. that ensures survival under extreme environmental conditions is their ability to deposit melanin within their cell wall and within the cytoplasm of arthroconidia, spherules and endospores, excluding fungal hyphae. Melanin deposition provides protection from extreme temperatures and ultraviolet radiation (UV) and, generally, from solar radiation [47, 48]. Gostinčar et al. [23] proposed a mechanism by which melanin may protect the fungus *Hortae werneckii* from high salinity. This fungus is able to synthesize 1, 8-dihydroxy-melanin as a protection from osmotic stress, which suggests that *Coccidioides* spp. that are also able to synthesize melanin may utilize similar protective mechanisms against hypersalinity. Additionally, *Coccidioides* produces hydrophobins that help this fungus to adapt and survive in their environment. It is interesting to speculate how these fungal structural components relate to different phenomena occurring at the surfaces that they encounter [49]. At the microorganism level, surface phenomena or conditions become the dominant force, whereas factors such as gravity are mostly insignificant. One of the functions of hydrophobins is to control surface forces [49]. When hyphae encounter aqueous surfaces, their surrounding hydrophobins provide a hydrophobic surface that helps in breaking the superficial tension, thus allowing the formation of micelles that separate from the aqueous media and travel through the air, increasing the efficiency by which conidia diffuse through the air [50].

The movement of humans and the migration of other mammals may serve as another mechanism for fungal dispersion in the environment [51]. An excellent example is the long distance migration of bats [52]. Work by Cordeiro et al. [33] demonstrated that these mammals are infected with *Coccidioides* spp., which may support the idea of bats as reservoirs and disseminators of this fungus. The transfer of animal species in captivity, for example, marine or terrestrial mammals from endemic zones that may be infected with *Coccidioides* [53] (Table 1), may serve as a mechanism for the dispersion of disease into non-endemic regions. These two forms of fungal dispersion may explain how transmission occurs in clinical cases within non-endemic zones, as those described in the state of Washington, where various cases of coccidioidomycosis have been reported, including one case of primary cutaneous coccidioidomycosis, two cases of pneumonia and one case that progressed to meningitis [54]. Furthermore, Litvintseva et al. [55] demonstrated persistent colonization of soils by *C. immitis* in Washington State to recent human infections [54] and confirmed genetic identity between isolates from soil and one of the case-patients.

This dispersal method would also provide an explanation for a positive *C. posadasii* isolate that was obtained from a patient who acquired the disease from a non-endemic region (Campeche, Mexico) and who reported no excursions away from his site of residence [25]. Another observation seldom referenced but with great relevance for the epidemiology of coccidioidomycosis is a report from Nigeria of two chickens that were analysed post-mortem and showed positive *Coccidioides* spp. isolates [56]. The relevance of this report lies in two specific elements: first, it represents the first report of coccidioidomycosis in the non-endemic region of Nigeria; second, it is the first report of the infection in birds, confirming that *Coccidioides* spp. are able to infect non-mammals. Another interesting case is that of a 14-year-old Chinese boy who had never visited or had been exposed to any imported materials from zones endemic for *Coccidioides* and yet was infected and suffered from coccidioidomycosis. Nevertheless, the boy was reported to have experienced an episode of drowning in the sea [57]. Although the source of the infection was unclear, it is known that *Coccidioides* can survive in salt water [18], and they are known to be present in marine mammals [58–60]. It is speculated that marine currents may be able to drag the arthroconidia towards non-endemic zones. These facts suggest that the area of geographic distribution for *Coccidioides* is expanding and that the fungi responsible for coccidioidomycosis are adapting to new habitats that may have characteristics different from those previously described [25].

Animal-to-animal transmission requires direct contact, such as handling infected animals at the clinic or direct inoculation with infected material [61], which occurs in an animal bite [62]. This concept is supported by the work of Lacy and Swatek [11] who reported on the finding of viable spherules in the tissue and secretion of animals.

There are various reports of coccidioidomycosis in domestic animals that have been confirmed by serologic evidence [63]. This poses a problem for public health because the presence of *Coccidioides* spp. in these animals suggests that they may serve as disseminators of the fungus and therefore be a possible danger to human health.

## Conclusions

The fungi that belong to the genus *Coccidioides* may be considered halotolerant organisms because they can survive and develop in environments with high salinity. These organisms can tolerate other environmental extreme conditions, such as high temperatures. To survive, these organisms rely on specific mechanisms that protect them against the damaging effects of extreme environmental conditions. Ultimately, life under extreme conditions may elicit changes in the reproduction, evolution, genetic makeup and speciation of these fungi. Therefore, it is important to gain knowledge about how *Coccidioides* spp. respond to stress and to understand how climate changes or human activity influence microbial diversity and evolution. To ensure their life cycle, *Coccidioides* spp. require animal reservoirs that probably serve as links with the environment by acting as disseminators of the fungi in nature.

## Abbreviations

ELISA: Enzyme-Linked ImmunoSorbent Assay
Meps: Metallo-proteases
PCR: Polymerase chain reaction
spp.: Species
USA: United States of America
UV: Ultraviolet radiation
